# Prevalence of Coronary Artery Calcification in Non‐Contrast Non ECG‐Gated Chest CT Scan of Patients With Significant Stenosis in Conventional Angiography in Comparison to Patients Without Significant Stenosis: A Cross‐Sectional Study

**DOI:** 10.1002/hsr2.71316

**Published:** 2025-09-29

**Authors:** Iman Abbaspour, Hoda Asefi, Abbas Soleimani, Golnaz Moradi, Samira Mirzaei

**Affiliations:** ^1^ Department of Radiology, Sina Hospital Tehran University of Medical Sciences Tehran Iran; ^2^ Department of cardiology, Sina Hospital Tehran University of Medical Sciences Tehran Iran

**Keywords:** coronary artery calcification, coronary artery stenosis, non‐contrast chest CT scan

## Abstract

**Background and Aims:**

Coronary artery disease is a main cause of mortality in developed and developing countries. Early diagnosis and treatment are important to reduce the burden of the disease. Correlation between coronary artery calcification in non‐contrast non‐ECG gated chest CT scan and conventional angiographic findings could be a helpful guide for risk assessment and the need for angiographic evaluation in patients with coronary artery calcification in non‐contrast chest CT scan applied for other reasons.

**Methods:**

A retrospective cross‐sectional study was conducted in the cardiology department of Sina hospital and patients who underwent angiographic study between march 2020 and april 2021 with recent non‐contrast non ECG‐gated chest CT scan were selected. patients with coronary stent and history of CABG were excluded and angiographic findings, prevalence, diameter and pattern of coronary artery calcification in CT scan was studied in the selected patients.

**Results:**

The prevalence of calcification in patients with significant stenosis was 94% and was significantly higher than patients without significant stenosis with prevalence of 46% (*p*‐value < 0.001). Calcification length and area in patients with significant stenosis was 67.1 and 218.4 mm^2^ and was significantly higher than patients without stenosis with length and area of 15.8 mm and 236.3 mm^2^ (*p*‐value < 0.001). there was meaningful correlation between length and area of calcification with maximum stenosis percentage seen in angiographic study (Pearson correlation: 0.61, 0.57).

**Conclusion:**

The presence and extent of CAC on non‐contrast, non‐ECG‐gated chest CT scans are correlated with significant coronary artery stenosis on angiography. These findings suggest that CAC assessment on routine chest CT scans can be used as a criterion for risk stratification and determining the need for angiographic evaluation to rule out significant stenosis.

## 1 Introduction

Cardiovascular disease remains a leading global health concern, responsible for more than 30% of all deaths, with coronary artery disease (CAD) contributing to approximately half of these fatalities [1]. The major cardiovascular disease risk factors identified globally include hypertension, smoking, diabetes, dyslipidemia, obesity, and physical inactivity, which together contribute significantly to the worldwide burden of cardiovascular morbidity and mortality [2, 3]. A significant proportion of patients with coronary artery disease (CAD), estimated at around 20%, present without conventional cardiovascular risk factors such as hypertension, hyperlipidemia, diabetes, or a history of smoking. This highlights the importance of developing diagnostic approaches capable of stratifying risk, especially in acute clinical environments [4]. Coronary artery calcium (CAC) imaging facilitates the identification of early, asymptomatic coronary atherosclerosis. Its presence in individuals without symptoms is linked to an increased likelihood of developing coronary heart disease (CHD) and experiencing higher overall mortality [5]. The presence and severity of coronary artery calcification (CAC) can be assessed using a non‐contrast chest CT scan. due to COVID‐19 pandemic, non‐contrast chest CT scan has been used as a diagnostic tool for evaluating extent of pulmonary involvement in emergency department. hence the increasing number of non‐contrast non‐ECG gated chest CT scan in emergency department, evaluating extent of coronary artery calcification can be used as an independent risk factor for coronary artery disease.

## 2 Methods and Materials

**Figure 1:**
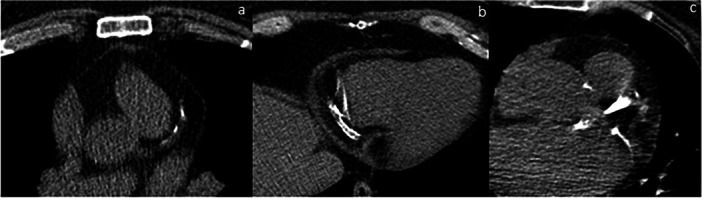
Calcification pattern studied visually in mediastinal window (WL:40, WW:350) (a) single layer: calcification presents in one arterial wall. (b) double layer: both arterial walls are calcified (c) confluent: circumferential arterial wall calcification is present so that the arterial lumen is not visible.

This single‐center retrospective cross‐sectional study was conducted in cardiology department of Sina hospital. Patients were enrolled between March 2020 and April 2021 provided by following inclusion criteria: patients underwent conventional angiographic evaluation in cardiology department and had recent non‐contrast non‐ECG gated chest CT scan within 2 months preceding or following angiography. Patients were excluded from the study if they had prior history of CABG or coronary artery stent placement. Patients were divided in two groups: first group was the patients who had significant stenosis in angiographic study. Patients without significant stenosis were included in the second group. Significant stenosis was defined as a more than 50% luminal stenosis in conventional angiographic study. Prevalence, diameter and pattern of coronary artery calcification (CAC) was studied in two groups and comparison was made for significant differences. A board‐certified radiologist with over 8 years of professional experience, reviewed all images manually for presence of coronary artery calcification in mediastinal window (WL:40, WW:350). Diameters of calcification was measured manually in 2 dimension similar to Agatston method: longitudinal measurement was done along the coronary artery, depending on the arterial pathway in axial plane and/or oblique reconstructed images along coronary artery axis and reported as calcification length. transverse measurement was done perpendicular to longitudinal axis and maximum transverse diameter was documented. With multiplying maximum transverse diameter by longitudinal diameter, calcification area was achieved and documented. Plaque density was manually measured multiple times along the plaque and maximum Hounsfield unit achieved, was documented. calcification area and length was studied for presence of correlation with the maximum luminal stenosis percentage reported in the angiographic study. Pattern of calcification was studied visually in mediastinal window (WL:40, WW:350), if calcification presents in one arterial wall, pattern was defined as single layer, if both arterial walls were calcified, pattern was defined as double layer, and if circumferential arterial wall calcification was present so that the arterial lumen was not visible, the term of confluent was used for documentation of pattern (Figure 1). The prevalence and diameter of calcification was also studied in men and women and comparison was made for significant differences. Also comparison was made between mean age of patients with coronary artery calcification and patients without calcification. Statistical analysis was performed using SPSS software, version 25 (IBM Corp). Quantitative data were summarized as means and standard deviations, while qualitative data were described using frequencies and percentages. Comparisons between the two groups were made using independent *t*‐tests. Associations between continuous variables were evaluated with Pearson's correlation coefficient. A *p*‐value of less than 0.05 was considered statistically significant for all analyses.

## 3 MDCT Protocols

All participants underwent CT imaging using a 16‐slice multidetector CT scanner (Somatom Sensation 16, Siemens Medical Solutions) in a single session without repositioning. The low‐dose ungated MDCT protocol included the following parameters: no ECG gating, a tube voltage of 110 kVp, and a tube current–time product of 80 mAs, utilizing automatic tube current modulation (Care Dose 4D, Siemens Medical Solutions). Additional settings were a rotation time of 0.6 s, detector collimation of 16 × 4 mm, a pitch of 1.5, and coverage spanning the entire lungs.

## 4 Results

The statistical analysis included 70 participants, evenly divided into two groups of 35. Among those with significant stenosis, there were 22 men and 13 women, with a mean age of 64.2 ± 9.5 years. In the group without significant stenosis, 21 men and 14 women were enrolled, with an average age of 54.5 ± 14.1 years. among 35 patients with significant stenosis, 33 patients had coronary artery calcification and CAC prevalence was 94.3% which was significantly higher in comparison to second group with CAC prevalence of 45.7% (16 of 35 patients) with *p*‐value of < 0.001. In the first group mean calcification length and mean calcification area for coronary arteries was 67.1 mm and 188.4 mm^2^ which was significantly higher than second group with calcification length and area of 15.8 mm and 36.3 mm^2^ (*p*‐value < 0.001). Mean of maximum Hounsfield Unit of calcification in the first group was 638 HU which was significantly higher than the second group with 443 HU (*p*‐value = 0.01). According to evaluation of CAC pattern and morphology, in 33 patients of first group with CAC, in 4 patients pattern was single layer, in 9 patients double layer and in 20 patients pattern was confluent. Among 16 patients with CAC in the second group, pattern in 6 patients was single layer, in 7 patients was double layer and in 3 patients was confluent. The confluent pattern had the highest prevalence in the first group (61%). In comparison, in the second group, confluent pattern had the lowest prevalence (19%) and the difference was significant (Pearson *χ* ^2^: 0.02). Summary of analyzes between two groups is concluded in Table 1.

**Table 1:** Variable analysis between two groups. Group A: patients with significant stenosis, group B: patients without significant stenosis, CAC: coronary artery calcification.

variable	Group A	Group B	Significance level
CAC prevalence	94%	46%	< 0.001
CAC length	67.1 mm	15.8 mm	< 0.001
CAC area	188.4 mm^2^	36.3 mm^2^	< 0.001
Maximum hounsfield unit	638 HU	443 HU	0.01
Confluent calcification pattern	61% (most common)	19% (least common)	0.02

Among 27 female patients that were included in this study, 21 patient had coronary artery calcification with prevalence of 78% which was not significantly different from male patients with CAC prevalence of 65% (28 of 43 male patients), (Pearson *χ* ^2^ = 0.26). calcification length and calcification area in male patients were 42 mm and 114 mm^2^ which was not significantly different when compared to female patients with calcification length and area of 41.1 mm and 111.3 mm^2^. (*p*‐value = 0.94, 0.94) Mean age of patients with coronary artery calcification was 64 years which was significantly older than patients without calcification with mean age of 48 years. (*p*‐value < 0.001).

## 5 Discussion

Series of studies has shown effectiveness of CAC score estimation for coronary artery disease [6]. In this study we used non ECG‐gated CT scan for the evaluation of coronary artery calcification, which is prone to cardiac motion artifact however, our study shows that prevalence of calcification in patients with significant stenosis in conventional angiography is higher than patients without significant stenosis, thus it is concluded that presence of CAC in non ECG‐gated CT scan is related to significant coronary artery disease. In study conducted by Gökdeniz et al between January 2012 and February 2013, a significant correlation was seen between CAC score and degree of stenosis seen in ECG‐gated CT angiography [7]. Our study also shows patients with significant stenosis had significantly higher calcification area and length, thus it is concluded that in addition to presence of calcification, extent of calcification seen in non ECG‐gated CT scan is also related with significant coronary artery disease.

The maximum calcification Hounsfield unit was also higher in patients with significant stenosis indicating the higher attenuation of calcified plaque, the higher risk of significant coronary artery disease.

Confluent pattern of calcification was the most common pattern observed in patients with significant stenosis and least common pattern in patients without significant stenosis, therefore it can be concluded this pattern of confluent plaque calcification is related with higher risk of significant coronary artery disease.

In prior studies as done by Kim et al., male patients had higher prevalence of coronary artery calcification and more extensive coronary artery atherosclerosis in comparison to female patients [8], In contrast, no meaningful difference was observed in our study regarding prevalence, length and area of calcification when comparison was made between male and female patients.

In a study conducted by Balakrishnan et al., between 1/1/2012 to 1/1/2013, patients with coronary artery calcification were older in comparison with patients without calcification [9]. Also in our study patients with coronary artery calcification were significantly older than the other group (mean age of 64 year vs. 48 years) indicating effects of aging on coronary artery atherosclerosis and calcification.

This study has some limitations that should be considered. The most important limitation is the relatively small sample size, with a total of 70 patients divided equally into two groups. While we used Cochran sample size formula for this study based on prevalence of coronary artery calcification in previous studies [10] and the results demonstrated statistically significant differences in the prevalence, extent, and pattern of coronary artery calcification between patients with and without significant coronary artery stenosis, the limited number of participants may affect the generalizability of these findings. Additionally, the retrospective, single‐center design of the study introduces the possibility of selection bias, as only patients who underwent both coronary angiography and recent noncontrast, non‐ECG‐gated chest CT scans were included. Another limitation is the use of non‐ECG‐gated chest CT, which, although practical and widely available, is prone to motion artifacts due to cardiac motion, potentially affecting the accuracy of coronary artery calcification detection and measurement. Despite this, the study demonstrated meaningful correlations with angiographic findings. Finally, although calcification patterns and extents were visually assessed by an experienced radiologist, the study did not incorporate interobserver variability analysis, which might have added robustness to the assessment of calcification patterns. Future research with a larger, multicenter cohort and prospective design is recommended to confirm these findings and further explore the diagnostic and prognostic value of coronary artery calcification assessment on non‐ECG‐gated chest CT scans.

## 6 Conclusion

Non‐ECG‐gated chest CT scans, despite their limitations, are valuable for assessing CAC and identifying patients at higher risk of significant coronary artery stenosis. These findings can guide risk stratification and the need for further angiographic evaluation.

## Author Contributions

**Iman Abbaspour:** investigation, writing – original draft, methodology, formal analysis, writing – review and editing, funding acquisition, validation, visualization, software, project administration, data curation, supervision, resources. **Hoda Asefi:** investigation, writing – original draft, supervision, data curation. **Abbas Soleimani:** conceptualization, writing – review and editing, supervision. **Golnaz Moradi:** supervision, writing – review and editing. **Samira Mirzaei:** supervision.

## Ethics Statement

Ethical approval for this study was obtained from the Research Ethics Committee of Sina Hospital, Tehran University of Medical Sciences (TUMS) (Approval ID: IR.TUMS.SINAHOSPITAL.REC.1400.085) and the requirement for informed consent was waived due to the retrospective nature of the study. All patient information remained confidential, and no personal identifiers were revealed. The study adhered to the ethical standards set forth in the Declaration of Helsinki.

## Conflicts of Interest

The authors declare no conflicts of interest.

## Transparency Statement

The lead author Iman Abbaspour affirms that this manuscript is an honest, accurate, and transparent account of the study being reported; that no important aspects of the study have been omitted; and that any discrepancies from the study as planned (and, if relevant, registered) have been explained.

## Data Availability

The data that support the findings of this study are available from the corresponding author upon reasonable request. Iman Abbaspour had unrestricted access to all study data and assumes full responsibility for their accuracy and integrity.
